# Aslanger Pattern: A Sign of an Acute Coronary Occlusion

**DOI:** 10.7759/cureus.84818

**Published:** 2025-05-26

**Authors:** Istria Barros, Alejandro Narvaez, Alberto Navarro, Carolina Cardona, Juan Senior

**Affiliations:** 1 Epidemiology, Universidad Del Rosario, Bogotá, COL; 2 Internal Medicine, Universidad de Antioquia Facultad de Medicina, Medellin, COL; 3 Interventional Cardiology, Hospital Universitario de San Vicente Fundación, Medellin, COL; 4 Interventional Cardiology, Universidad de Antioquía, Medellín, COL; 5 Cardiology, Hospital Universitario de San Vicente Fundación, Medellin, COL; 6 Cardiology, Universidad de Antioquía, Medellín, COL; 7 Interventional Cardiology, Universidad de Antioquía, Medellin, COL; 8 Interventional Cardiology, Hospital Universitario de San Vicente Fundacion, Medellin, COL

**Keywords:** acute chest discomfort, acute myocardial infarction, aslanger pattern, st elevation, unusual causes of chest

## Abstract

ST-elevation criteria miss a substantial number of acute coronary occlusions, resulting in treatment delays and worse prognosis. The Aslanger pattern has been proposed as a new pattern that, despite not meeting the definition of ST-elevation myocardial infarction, represents an acute coronary occlusion. Therefore, patients with this pattern could benefit from early revascularization. The case of a man with acute chest pain is presented, whose initial electrocardiogram showed an Aslanger pattern. Due to the misdiagnosis at the primary care center and the emergency room, the patient did not receive timely optimal management. This case remarks the importance of recognition of this new pattern and its impact on decision-making in patients with acute coronary syndrome.

## Introduction

ST elevation is a key finding in the diagnosis of acute myocardial infarction (AMI). However, this criterion neglects more than a quarter of acute coronary occlusions, resulting in treatment delays [1]. Therefore, it is important to recognize new electrocardiographic patterns that address these shortcomings. In 2020, Aslanger et al. published a pattern that was associated with acute coronary occlusion and multivessel disease, although it did not meet the traditional definition of STEMI. Recognizing this pattern could allow for early revascularization and thus improve patient prognosis. The ECG pattern was defined as: (1) any ST elevation present in lead DIII but absent in other inferior leads; (2) ST depression in any of leads V4 to V6, with a positive or terminally positive T-wave; and (3) ST elevation in lead V1 higher than that in lead V2 [2]. In recent years, some authors have suggested changing the definition of acute coronary syndromes by proposing the term “occlusion myocardial infarction (OMI)”, which includes not only patients with STE but also those with other patterns associated with acute occlusion, that is, those who may benefit from early revascularization. These include the Aslanger pattern, the precordial swirl, hyperacute T waves, ST depression in V1 to V3, or the South African flag pattern [3].

## Case presentation

Case description and narrative review

A 70-year-old man living in a rural area, with a history of active smoking, presented to the primary care center with acute chest pain that had started eight hours earlier. The pain radiated to his jaw and was described as a tight, constricting sensation. He was transferred to a quaternary care hospital, where he arrived 36 hours after the onset of symptoms. At the time of the interview, he reported no recurrence of chest pain. At physical examination, blood pressure was 110/60 mmHg, heart rate was 75 bpm, respiratory rate was 18 breaths/min, and oxygen saturation was 95%. He had no remarkable findings in the rest of the physical exam. Serum high-sensitive troponin I (Atellica) was 11.036 ng/L (reference range: 0-40 ng/L), and his GRACE (Global Registry of Acute Coronary Events) score was 134 points. In Table 1, we present the laboratory findings, and the timeline is shown in Table 2.

**Table 1:** Laboratory findings

Laboratory Parameters	Patient Results	Reference Range
White blood cells (mm^3^)	7.340	4.500–11.000
Lymphocytes (mm^3^)	1.208	1.000–4.500
Neutrophils (mm^3^)	5.435	1.800–7.700
Hemoglobin (g/dl)	13.5	13.5–17.5
Hematocrit (%)	48	41–53
Platelets (mm^3^)	235.700	150.000–450.000
Thromboplastin time (seconds)	28.1	25–35
Prothrombin time (seconds)	12.8	11–13.5
Creatinine (mg/dl)	1.2	0.50–0.97
Troponin I high-sensitive ng/L (Atellica)	11.036	0–40

**Table 2:** Timeline AVR: Augmented vector right; ECG: Electrocardiogram; GRACE: Global Registry of Acute Coronary Events; LAD: Left anterior descending artery; LCX: Left circumflex artery; LVEF: Left ventricular ejection fraction; NSTE-ACS: Non-ST elevation acute coronary syndrome; NYHA: New York Heart Association; PCI: Percutaneous coronary intervention; RCA: Right coronary artery; TTE: Transthoracic echocardiogram.

Timeline	
Day 0	Onset of symptoms
Day 1	The patient presents to a primary care center.
	Initial evaluation is done.
	Referred to a quaternary care hospital.
Day 2	Arrival at quaternary care hospital.
	No recurrence of pain at the time of the interview.
	hs-troponin I: 11,036 ng/L (markedly elevated).
	GRACE score: 134 (intermediate risk).
	Initial ECG showed sinus rhythm, P wave inversion (inferior leads), positive AVR, ST elevation in V1 > V2 and III, Q wave in III, ST depression in I and aVL, and suspected ectopic atrial rhythm.
	Initially classified as NSTE-ACS.
Day 3	Coronary angiography performed.
	RCA: Total occlusion (culprit artery).
	LAD: 80% severe stenosis (proximal-mid).
	LCX: Complex severe proximal lesion.
	Diagnosis of the Aslanger pattern was confirmed by an interventional cardiologist.
	Intervention: RCA and LCX not intervened (>48 h + risk).
	LAD: Balloon angioplasty + stent.
Post-PCI	ECG shows a return to sinus rhythm and confirms transient ectopic atrial rhythm.
	TTE: LVEF 45%, inferior wall akinesia.
Day 5	Discharged with dual antiplatelet therapy (DAPT), statins, and beta-blockers.
Day 180	Follow-up. NYHA class I.
	No recurrence of chest pain.
	No complications.
	No further interventions required.

**Figure 1:**
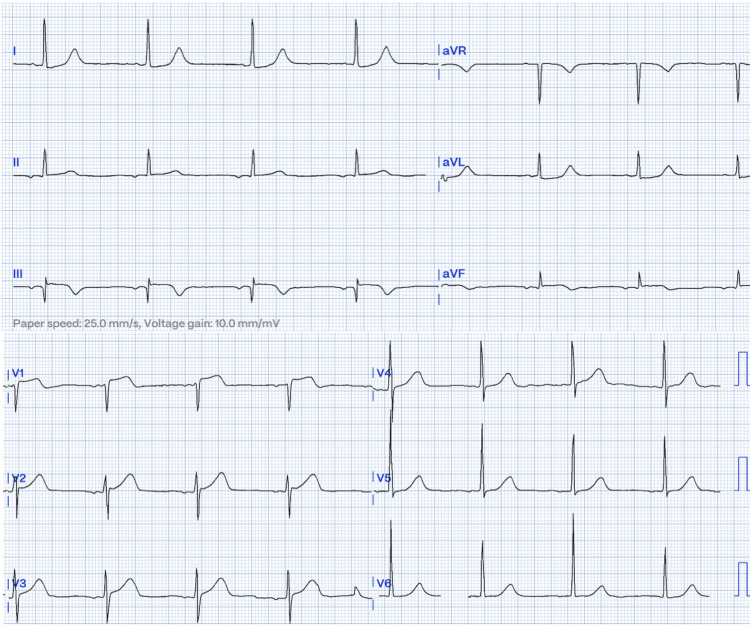
Initial ECG: Sinus rhythm; HR: 49 bpm; negative p wave in the inferior leads, positive in AVR; ST elevation in III and V1, greater in V1 than V2; Q wave in III; ST depression in I, aVL, V5, and V6; inverted T waves in III and AVF. Note: In the original ECG, there was a subtle ST deviation in V5 and V6, but after improving the quality by digitizing, it is not observed in the presented image.

The initial electrocardiogram (ECG) showed sinus rhythm, with P wave inversion in the inferior wall leads and positive AVR, ST elevation in leads V1 and III, with greater elevation of the ST segment in lead V1 than V2, Q wave in III, and ST depression in leads I, aVL. In the original ECG, there was a subtle ST deviation in V5 and V6, but after improving the quality by digitizing, it is not observed in the presented image (Figure 1).

Initially, the patient was approached using the non-ST elevation acute coronary syndrome (NSTE-ACS) pathway; therefore, coronary angiography was planned in the first 24 hours. Additionally, an ectopic atrial rhythm was suspected. When the patient arrived at the catheterization laboratory, the interventional cardiologist reviewed the initial ECG, and an Aslanger pattern was diagnosed based on the findings.

**Figure 2:**
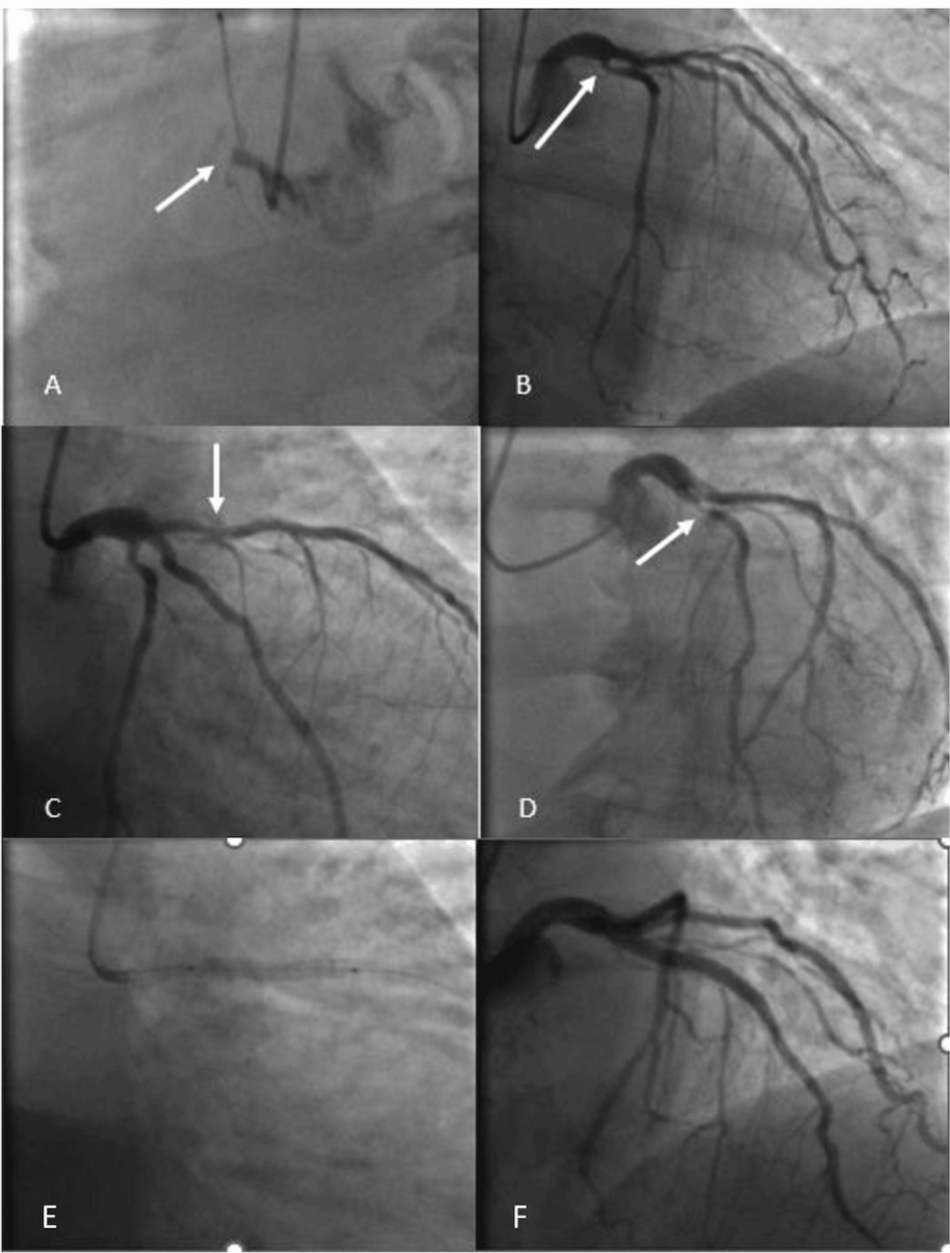
(A) Occluded right coronary artery. (B) Circumflex coronary artery with a 70% proximal lesion. (C) and (D) Left anterior descending coronary artery with a severe lesion in the proximal third. (E) and (F) Successful angioplasty and stent placement in the left anterior descending coronary artery.

The coronary angiography was performed 50 hours after the onset of the pain, which showed complete occlusion of the right coronary artery (RCA, culprit artery), a severe occlusion of 80% in the proximal to medial segment of the left anterior descending artery (LAD), and a severe and complex lesion in the proximal segment of the circumflex artery (LCX) (Figure 2, Panels a-d). Due to the duration of symptoms (>48 hours), no intervention was performed on the RCA and LCX, and medical management was offered. The patient had declined surgical revascularization; hence, only the LAD was intervened with balloon angioplasty and stent placement (Figure 2, Panels e and f).

**Figure 3:**
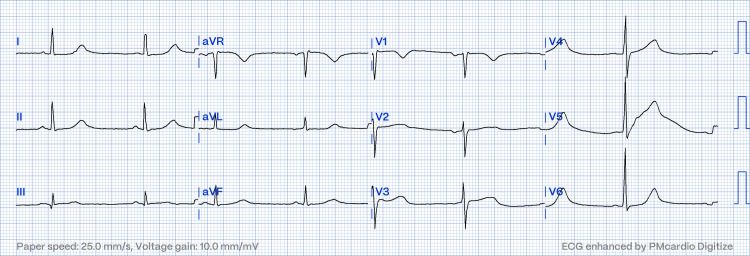
Follow-up ECG after checking the extremity leads: sinus bradycardia, HR: 52 bpm, and qR in III

Due to technical aspects and the risks of the intervention, LCX intervention was not performed. Follow-up ECG showed restoration of sinus rhythm, confirming the transient ectopic atrial rhythm (Figure 3). The patient had a favorable clinical course, and the transthoracic echocardiogram reported a left ventricular ejection fraction of 45% with inferior wall akinesia. He was discharged 48 hours after admission with double antiplatelet therapy, statins, and beta-blockers. At the six-month follow-up, clinical evolution was favorable; he was stable with a New York Heart Association (NYHA) functional class I status, no recurrence of the chest pain, no other complications, and no need for further interventions.

## Discussion

We present a case of a man with acute chest pain whose initial ECG showed an Aslanger pattern, and the patient did not receive timely optimal management due to his delayed recognition. Considering the above, patients with this pattern should undergo emergent revascularization, as it represents an OMI. This is of great relevance, as it has been proven that up to 27% of infarctions classified as NSTE-ACS really have an occlusive infarction. These lesions are frequently localized in arteries that irrigate the inferolateral wall and are associated with larger infarct zones and greater mortality [1].

In 2020, Aslanger et al. described a new electrocardiographic pattern associated with acute occlusion of the RCA or LCX in a population of 1000 patients with NSTE-ACS [2]. The authors proposed three criteria: (a) elevation of the ST segment present in lead III but absent in II or AVF; (b) ST depression in any of leads V4-V6, with positive T-wave; and (c) ST elevation in lead V1 greater than that in V2. According to current guidelines [3], this pattern is considered in NSTE-ACS; however, patients with this electrocardiographic pattern can indicate occlusive coronary disease involving the inferior wall, with at least one critical stable coronary lesion in the rest of the arteries not related to the infarction artery. In the original population, 6.3% of patients initially diagnosed with NSTE-ACS presented this pattern, in which the culprit artery was LCX in 50% of cases and RCA in 32% of cases. These patients had larger infarcts, multivessel disease, multiple comorbidities, and greater baseline risk, but they had similar short- and long-term outcomes compared to other patients with NSTE-ACS [2].

The explanation for this atypical pattern seems to be due to the ST vector not being directed toward the injured zone in the inferior wall. Apparently, the ST vector is headed more rightward than usual due to the sum of potentials of the ST vector in the inferior wall and the vector of the ischemic subendocardium, generated by the critical stenosis of the accompanying vessel. The sum of these two vectors results in a vector directed to the right, projecting itself not only to the negative pole of lead II but also to the positive pole of III. In contrast, the direction of the vector from the ischemic subendocardium does not localize the ischemic area and always points toward the positive pole of AVR, regardless of the involved territory. As a result, the electrocardiogram shows an ST elevation in III and AVR, coupled with an ST deviation in I, AVL, and V4-V6, while remaining isoelectric in AVF [2].

This pattern can be confused with the electrocardiographic changes of multivessel disease or the occlusion of the main left coronary artery occlusion. These two conditions have ST elevation in AVR and diffuse ST segment depression; however, unlike the Aslanger pattern, they do not have ST elevation in III [4,5]. Despite AVR not being considered an inferior lead, there is a 90° angle between AVR and III in the frontal plane; so, they are contiguous anatomically. Additionally, according to the concept published by Litmann in 2021, leads V1-V2-V3 could have a manifestation in the frontal plane, and their morphology is similar to AVR, aVL, and AVF, respectively. Analyzing the Aslanger pattern under the Litmann perspective, despite not being continuous, ST elevation occurs in related leads: III, AVR, and V1 [6].

Furthermore, this case underscores the importance of early identification of the pattern. If it had been diagnosed in time, the patient would have been quickly transferred to the catheterization laboratory, and the culprit vessel would have been reperfused as the greatest benefit occurs if revascularization is performed in the first 12 hours. The current guidelines recommend against the routine percutaneous coronary intervention of the culprit artery in ST-elevation myocardial infarction patients presenting more than 48 hours after the onset of symptoms and without persistent symptoms [7].

Curiously, the present case initially showed sinus bradycardia, a low ectopic atrial rhythm, sinus bradycardia, and junctional escape beats. Rhythm disturbances in ACS are common [8]. The pathogenesis is not known, although several possible mechanisms have been postulated, including ischemia due to the release of products of tissue breakdowns and either direct or reflex cholinergic suppression of sinus node activity. Most are transient and reversible even in the absence of reperfusion, possibly related to the sinoatrial node susceptibility to ischemia and resistance to necrosis. Although certain atrial rhythms can mimic an inferior wall ST-elevation myocardial infarction, the transient evolution and the findings in the coronary angiography suggest that it represents an epiphenomenon of sinus node ischemia, explained by the nodal irrigation through the branches of the RCA and LCX [9,10].

## Conclusions

The Aslanger pattern, despite not fulfilling the standard definition of ST elevation, represents an acute occlusion of the RCA and LCX, with multivessel disease. This specific electrocardiographic pattern consists of elevation of the ST segment in lead III, but not in II or AVF, ST depression in V4-V6 with positive T waves, and ST elevation in lead V1 greater than that in V2.

This ECG pattern is suggestive of acute coronary occlusion involving the inferior wall, with at least one critical stable coronary lesion in the rest of the arteries not related to the infarction artery. Early identification will impact decision-making in patients with the Aslanger pattern, leading to rapid revascularization and an even better prognosis.
